# A Gamified Smartphone-Based Intervention for Depression: Randomized Controlled Pilot Trial

**DOI:** 10.2196/16643

**Published:** 2021-07-20

**Authors:** Christian Aljoscha Lukas, Bjoern Eskofier, Matthias Berking

**Affiliations:** 1 Department of Clinical Psychology and Psychotherapy Friedrich-Alexander-University Erlangen-Nuremberg Erlangen Germany; 2 Department of Computer Science Friedrich-Alexander-University Erlangen-Nuremberg Erlangen Germany

**Keywords:** smartphone technology, depression, cognitive behavioral therapy, approach/avoidance, gamification

## Abstract

**Background:**

Available smartphone-based interventions for depression predominantly use evidence-based strategies from cognitive-behavioral therapy (CBT), but patient engagement and reported effect sizes are small. Recently, studies have demonstrated that smartphone-based interventions combining CBT with gamified approach-avoidance bias modification training (AAMT) can foster patient engagement and reduce symptoms of several mental health problems.

**Objective:**

Based on these findings, we developed a gamified smartphone-based intervention, mentalis Phoenix (MT-Phoenix), and hypothesized the program would both engage patients and produce preliminary evidence for the reduction of depressive symptoms.

**Methods:**

To test this hypothesis, we evaluated MT-Phoenix in a randomized controlled pilot trial including 77 individuals with elevated depression scores (Patient Health Questionnaire-9 scores ≥5). Participants were either instructed to train for 14 days with MT-Phoenix or assigned to a waitlist control condition. Engagement with the intervention was measured by assessing usage data. The primary outcome was reduction in depressive symptom severity at postassessment.

**Results:**

Data from this pilot trial shows that participants in the intervention group used the smartphone-based intervention for 46% of all days (6.4/14) and reported a significantly greater reduction of depressive symptoms than did participants in the control condition (*F*_1,74_=19.34; *P*=.001), with a large effect size (*d*=1.02). Effects were sustained at a 3-month follow-up.

**Conclusions:**

A gamified smartphone-based intervention combining CBT with AAMT may foster patient engagement and effectively target depressive symptoms. Future studies should evaluate the effectiveness of this intervention in a phase 3 trial using clinical samples. Moreover, the intervention should be compared to active control conditions.

**Trial Registration:**

German Clinical Trial Registry DRKS00012769; https://tinyurl.com/47mw8du7

## Introduction

Depression is one of the most common mental disorders in the world [1] and is associated with severe impairments for afflicted individuals [2]. Fortunately, there is ample evidence for the efficacy of psychotherapeutic treatments for depression [3]. However, in spite of costly attempts to provide evidence-based treatment for all patients, a large number of individuals remain untreated [4,5] or respond to psychotherapy only partially [6].

In the past decade, many attempts have been made to use the internet to improve access and response to depression treatments. Due to the dramatic increase in smartphone use in the past years [7], these attempts have recently started to focus on smartphone-based interventions for mental health [8]. This focus can be explained by the advantages of smartphones such as their widespread use [9], their resulting potential for dissemination [10], and their constant availability, which allows for the integration of therapy-relevant competencies into the patient’s daily life [11].

Available research shows that smartphone-based interventions using strategies from cognitive-behavioral therapy (CBT) have the potential to effectively reduce depressive symptoms. For example, a study [12] showed that a smartphone-based intervention offering 6 weeks of self-guided CBT for adults with mild-to-moderate depression led to significant reductions of depressive symptoms when compared to a control condition with access to internet-based psychoeducation for depression (*d*=1.03). Significant effects were also found on measures of behavioral activation and work-related outcomes such as productivity, absence rates, and workplace distress. In another trial [13], participants from a community sample with mild-to-moderate symptoms of depression received 7 weeks of training with a CBT-based self-help smartphone-based intervention. At postassessment, participants in the intervention group showed significantly greater reductions of depressive symptoms than both an attention control group (*d*=0.36) and a waitlist control condition (*d*=0.46).

However, results for the effectiveness of CBT-based smartphone-based interventions for depression are inconsistent. For example, a three-armed trial [14] compared 4 weeks of treatment with a gamified smartphone-based intervention that targets cognitive control abilities to a smartphone-based intervention based on problem-solving therapy and an information control smartphone-based intervention in a sample of participants with mild-to-moderate symptoms of depression. Although depression symptoms decreased in the total sample, no significant differences were found between the two active smartphone-based interventions and the information control condition at both 4-week and 12-week follow-up assessment. Moreover, meta-analysis results suggest that heterogeneous smartphone-based interventions for depression are moderately effective when compared to inactive controls (*g*=0.56), but that effects are small when compared to active control conditions (*g*=0.22) [15]. In the meta-analysis, a subgroup analysis showed that the use of CBT techniques did not influence study effect sizes significantly when compared to smartphone-based interventions that did not use CBT.

In addition to smartphone apps using techniques from CBT, smartphone-based interventions using cognitive bias modification (CBM) paradigms have recently been discussed as potentially useful for the reduction of symptoms in various psychological domains. For depression, available CBM interventions have focused primarily on the modification of attention biases [16] and interpretation biases [17]. Regarding their effectiveness, a meta-analysis study reported a moderate effect of attention bias modification and interpretation bias modification trainings on biases (*g*=0.49), but only a small effect on anxiety and depression symptoms (*g*=0.13) [18]. Lately, approach-avoidance biases have been shown to play an important role in the development and maintenance of depression. For example, one study showed that depressed individuals have a stronger avoidance tendency toward angry faces when compared to healthy controls (*d*=0.26) [19]. In another study [20], the authors found reduced approach motivation toward positive pictures compared to neutral pictures in individuals with depressive symptoms when compared to nondepressed controls (*d*=0.55). In trainings aiming at the modification of approach-avoidance biases (approach-avoidance modification training [AAMT]), participants are asked to approach functional disorder-specific stimulus material (pictures and/or statements) and to avoid dysfunctional material. Prominent examples for the clinical utilization of computer-based AAMT can be found in the domain of alcohol dependency. Here, two studies demonstrated that the combination of 3 months of inpatient CBT and computer-based AAMT effectively reduced relapse rate in alcohol-dependent individuals after a 12-month follow-up by 10% [21] to 13% [22] when compared to CBT-only controls. In the domain of depression, a recent study used computerized AAMT as an add-on to treatment-as-usual [23]. In a sample of clinically depressed individuals, this blended intervention was shown to successfully reduce depressive symptoms when compared to a sham control condition. Another study included patients with various diagnoses and tested AAMT as an adjunct to inpatient treatment [24]. Here, results showed that AAMT reduced depressive symptoms compared to a sham control group. Interestingly, approach tendencies and symptom reductions were moderated by depression severity at baseline, such that only participants with higher initial depressive symptoms benefited from this intervention.

With regard to smartphone-based interventions facilitating a similar blended approach, pilot studies yielded promising results for the efficacy of gamified smartphone-based interventions combining AAMT with face-to-face CBT in various psychological domains. In one of these studies, the combination of 14 days of gamified AAMT with a brief face-to-face counseling session led to significant reductions in body dissatisfaction in individuals at risk for eating disorders when compared to waitlist controls (*d*=0.62) [25]. In another study, two brief face-to-face group counseling sessions and 14 days of gamified AAMT significantly reduced procrastination when compared to a waitlist control condition (*d*=0.84) [26]. A third study tested the aforementioned rationale in the domain of alexithymia and showed the intervention significantly reduced alexithymia (*d*=1.14) and improved emotion recognition skills (*d*=0.97) when compared to an active control condition [27].

Aside from the utilization of psychotherapeutic techniques, some studies have suggested that low adherence rates to smartphone-based interventions and the lack of engagement features used by apps may be partly responsible for the limited effectiveness of available interventions [28]. In search of ways to improve patient engagement, several studies [29,30] have discussed the use of gamification elements as a particularly promising tool to increase engagement in nongaming contexts. Gamification refers to the use of game elements and design features such as points, badges, levels, progress, and challenges in nongaming software [31]. Regarding the use of gamification strategies to increase engagement with online interventions, a systematic review demonstrated that gamification has the potential to increase engagement parameters such as time spent in a program, number of completed assignments, and total number of views [32]. In depression research, a meta-analysis that analyzed gamified interventions targeting depression found a moderate effect size for depression therapy at posttreatment (*d*=0.47) [33].

Addressing the important role of CBT and approach-avoidance biases in depression as described above and following up on the promising findings for smartphone-based interventions that combine gamified AAMT with face-to-face CBT in other domains, we developed an automated, gamified smartphone-based intervention for depression combining AAMT with CBT (mentalis Phoenix [MT-Phoenix]). To provide a scalable and possibly cost-effective intervention, we developed MT-Phoenix as a standalone smartphone-based intervention. The aim of this study was to test MT-Phoenix in a phase 2 randomized controlled pilot trial to explore the effectiveness of this novel intervention in a cohort of individuals with elevated depressive symptoms. We hypothesized MT-Phoenix would reduce depressive symptoms and improve well-being. Treatment effects were expected to be stable at a 3-month follow-up.

## Methods

### Recruitment

Participant recruitment started in May 2017 through announcements published on the internet (ie, across several social media channels and local notice boards). Interested individuals were asked to scan a QR code or click a link provided in the announcements that led to a survey tool (Unipark) providing a screening questionnaire that assessed participants for study inclusion based on the following criteria: heightened depression scores with values ≥5 on the Patient Health Questionnaire-9 (PHQ-9) [34], sufficient German language skills, aged ≥18 years, access to a smartphone using iOS (Apple iPhone 5 or above), and ability to provide informed consent. Eligible individuals were sent written information about study procedures and an informed consent form via email. Participants that returned a signed copy of the informed consent form were randomly assigned to either the intervention or a waitlist control condition. We used block randomization with a fixed block size of two to ensure similar sample sizes across conditions. Randomization was conducted by a master’s degree student (not otherwise involved in the study) using a randomization website. Participants received an email with a link to the survey tool reminding them to complete both the primary and secondary outcome measures. Posttreatment assessment was conducted 2 weeks after baseline, follow-up assessment was conducted 12 weeks after posttreatment assessment. The treatment was free of charge. Student participants received course credit for participation and every participant automatically took part in a draw for a shopping gift card. All data were assessed with the help of the survey tool. After baseline completion, participants in the intervention group received an email inviting them to download MT-Phoenix in the App Store and to train over a 14-day period. Given the heterogeneity of studies in this emerging field, no standardized recommendations on the use of smartphone-based interventions have come to the authors’ attention. Thus, participants did not receive any recommendations regarding duration or frequency of use of the intervention in this pilot trial. Participants in the waitlist condition were given access to the intervention after completing the follow-up assessment. All study procedures complied with the human research guidelines of the Declaration of Helsinki and were approved by the ethics committee of the German Psychological Society.

### Intervention

#### Overview

MT-Phoenix was developed by a graduate psychologist (CAL) and a professor in clinical psychology (MB). MT-Phoenix is a gamified intervention that provides the trainings for 13 module-based competencies important for managing depressive symptoms. Multimedia Appendix 1 shows screenshots of the app. The 13 competencies in MT-Phoenix are presented in this order to the participants: functional thoughts, positive activities, daily routines, experiencing pleasure, relaxation, reconnect socially, self-support, self-comfort, problem-solving, acceptance, grieving, gratitude, and self-care. Gamification is used by arranging the modules sequentially, having participants earn points for completion of certain activities, using a level system in the AAMT, and providing illustrated feedback components. The competencies consist of the elements described below.

#### Psychoeducation

At the start of each competency module, MT-Phoenix provides information on the relevance of the respective competency for depression. Consistent with typical smartphone use, educative information is provided by simulating a fictional group chat in which a coach and four users affected by depression communicate with each other via made-up SMS text messages. In the fictional conversations, the coach makes use of two conventional therapeutic techniques: guided discovery and Socratic dialogue.

#### AAMT/Audio Instructions

In 10 of the 13 competencies, MT-Phoenix uses four different types of gamified AAMT in which participants are asked to systematically approach functional stimulus material and avoid dysfunctional stimulus material. Approach-avoidance is achieved by making use of the smartphone’s several input channels. Three consecutive levels of AAMT are provided in each of the 10 competencies containing AAMT. Before the start of a new level, MT-Phoenix provides a short tutorial on how the respective level is played. Made-up stimulus material was provided for each competency. The stimuli used in the AAMT are competency-specific pictures (faces, scenes, etc) with text statements (negative thoughts, dysfunctional beliefs, etc) written on them. Examples of stimuli can be found in Multimedia Appendix 2. In the first level (SWIPE), approach-avoidance is trained by asking participants to wipe away dysfunctional stimuli and to pull functional stimuli toward themselves by moving the stimulus either to the top or the bottom of their smartphone screen with their finger. In the second level (COMMAND), participants can control the stimuli with voice commands (eg, saying the words “future” or “friend” to a functional stimulus, making it move toward oneself, and the words “past” or “foe” to a dysfunctional stimulus, making it disappear from the smartphone display) by making use of the smartphone’s microphone. In the third level (DRAW), participants are asked to approach or avoid stimuli by drawing meaningful gestures (eg, by drawing a check mark on a functional stimulus or by “crossing out” a dysfunctional stimulus) on the smartphone display. Here, approach-avoidance is reinforced by making checkmarks appear in green and crosses in red. In the fourth level (MAZE), the stimuli are placed in the center of different labyrinths and participants are instructed to maneuver stimuli through the labyrinths before pulling functional stimuli toward themselves or wiping away dysfunctional stimuli. Upon correct and incorrect reactions, MT-Phoenix provides feedback (positive feedback: showing illustrated thumbs-up pictures and the word “Correct!”; negative feedback: illustrated thumbs-down pictures, the words “That’s wrong!” and a short vibration of the smartphone) to the participants. For three competencies (relaxation, experiencing pleasure, and self-support), MT-Phoenix provides audio instructions instead of AAMT.

#### Tasks

At the end of each competency module, participants are asked to complete a series of competency-related short tasks. Short tasks are exercises designed to foster motivation and behavioral activation (eg, “Go for a 15-min walk today,” “Try to think of three things you liked today and write them down in the app”). Studies have found behavioral activation tasks in smartphone apps for depression to be particularly helpful [35]. Participants have to complete a minimum of three short tasks to successfully “play through” a competency and to continue to the subsequent module in the training.

### Measures

#### Primary Outcome

Depressive symptoms were assessed using the PHQ-9 [34]. The PHQ-9 is a 9-item self-report questionnaire that evaluates the presence of depressive symptoms during the last 14 days based on the Diagnostic and Statistical Manual of Mental Disorders, 4th Edition (DSM-IV) diagnostic criteria for major depression. Each of the 9 items can be scored from 0 (not at all) to 3 (nearly every day) so that scores can range from 0 (absence of depressive symptoms) to 27 (severe depressive symptoms). The German version of the PHQ-9 used in this study has been shown to have high sensitivity (95%) and specificity (86%) in the detection of depression [36]. In previous studies, the internal consistency of the PHQ-9 has been demonstrated as good, with an α score ranging from .86 to .89. In this study, the α for the PHQ-9 was .86.

#### Secondary Outcomes

Presence of emotional, motivational, cognitive, somatic, and interactional aspects of depression during the last 7 days was assessed with the German 20-item version of the Center for Epidemiological Studies Depression Scale (Allgemeine Depressions-Skala [ADS]) [37]. Higher values indicate more severe depressive symptoms. Internal consistency has been demonstrated as good, with α scores ranging from .89 to .92. In this study, the internal consistency was .91.

Well-being was assessed using the 5-item World Health Organization Well-being Index (WHO-5) [38]. On a 5-point Likert-type scale ranging from 0 (none of the time) to 5 (all of the time), the WHO-5 asks respondents to rate how the following statements applied to them during the last 14 days: “I have felt cheerful and in good spirits,” “I have felt calm and relaxed,” “I have felt active and vigorous,” “I woke up feeling fresh and rested,” and “My daily life has been filled with things that interest me.” Internal validity has been shown to be excellent (Cronbach α=.92) in a German-speaking sample. The α for this study was .89.

### Statistical Analysis

Possible intervention effects were evaluated using an intention-to-treat approach. Missing data were shown to be missing completely at random (nonsignificant Little test), imputed with the help of Markov chain Monte Carlo multivariate imputation algorithm with 10 estimations per missing value [39]. We conducted analyses of covariance (ANCOVAs) on post and follow-up outcome scores to evaluate possible intervention effects and included the outcomes’ baseline values as covariates to control for a potentially confounding influence of these scores. As for effect sizes, we calculated Cohen *d* based on [40] and followed commonly used conventions [40] by defining 0.20, 0.50, and 0.80 as small, moderate, and large effects, respectively.

## Results

### Participants

Multimedia Appendix 3 illustrates the flow of participants through the study. In the final sample of 77 participants, the mean age was 29.93 (SD 11.61) years. Participants were predominately female (56/77, 82%) and 34% (26/77) of participants reported they were receiving therapeutic treatment at the time. Significant baseline differences between the intervention and the control condition were found with regard to depression measured with the PHQ-9, age, and occupation. Table 1 displays sociodemographic and clinical characteristics at baseline.

**Table 1:** Sociodemographic and clinical characteristics of participants at baseline.

Variable		Intervention group (n=40)	Control group (n=37)	Test type	*P* value
Age, mean (SD)		35.05 (13.64)	24.25 (4.31)	Wilcoxon rank-sum test	.001
Gender (female), n (%)		31 (82)	25 (86)	Fisher exact test	.74
**Country of origin, n (%)**					
	Germany	38 (95)	37 (100)	Fisher exact test	>.99
	Austria	1 (5)	0 (0)		
**Occupation, n (%)**					
	Student	14 (35)	27 (73)	Fisher exact test	.02
	Employed	19 (48)	4 (11)		
	Unemployed	2 (5)	1 (3)		
	Other	5 (12)	5 (14)		
**Education, n (%)**					
	<10 years	8 (20)	5 (14)	Fisher exact test	.54
	>10 years	32 (80)	32 (86)	Fisher exact test	.53
Psychotherapy (no), n (%)		22 (55)	29 (78)	Fisher exact test	.048

### Intervention Effects

ANCOVA results on depressive symptoms as assessed with the PHQ-9 revealed significant differences between the intervention and the waitlist control condition at postintervention assessment (*F*_1,74_=19.34; *P*=.001) with a large effect (*d*=1.02). Of all treated participants, 63% (25/40) achieved clinically significant improvement on the primary outcome measure as defined by a reduction ≥5 points on the PHQ-9. Regarding depressive symptoms as assessed with the ADS, the ANCOVA yielded significant differences between the intervention and the waitlist control condition after the intervention (*F*_1,74_=36.68; *P*=.001) with a large effect (*d*=1.41). With regard to well-being, ANCOVA results showed significant differences between the intervention and the waitlist control condition at postintervention assessment (*F*_1,74_=15.34; *P*=.001) with a large effect (*d*=0.91). Empirical means and standard deviations are displayed in Table 2.

**Table 2:** Means and standard deviations for primary and secondary outcomes.

Outcomes	Intervention group (n=40)			Control group (n=37)		
	Pretreatment, mean (SD)	Posttreatment, mean (SD)	Follow-up, mean (SD)	Pretreatment, mean (SD)	Posttreatment, mean (SD)	Follow-up, mean (SD)
Primary outcome: Patient Health Questionnaire (PHQ-9)	11.65 (5.27)	8.11 (4.07)	9.55 (4.64)	14.87 (4.43)	13.48 (4.69)	13.79 (4.85)
Secondary outcome: General Depression Scale (ADS)	48.35 (10.88)	39.91 (7.95)	43.39 (11.87)	51.47 (7.79)	50.88 (8.65)	50.40 (9.74)
Secondary outcome: World Health Organization Well-being Index (WHO-5)	7.77 (4.50)	10.76 (4.25)	10.57 (4.09)	7.67 (3.09)	7.75 (3.37)	7.91 (3.57)

### Maintenance of Effects

Regarding the maintenance of treatment effects, ANCOVA results showed that intervention effects persisted through follow-up for the PHQ-9 (*F*_1,74_=6.35; *P*=.014), the ADS (*F*_1,74_=5.85; *P*=.018), and the WHO-5 (*F*_1,74_=14.72; *P*=.001).

### Intervention Engagement and Evaluation

With regard to intervention engagement, 13 participants did not initiate training with the intervention over the intervention period. The 27 participants that did initiate training with MT-Phoenix used the app for an average of 6.38 days (SD 2.83) and spent 62.39 minutes in the app (SD 68.17). During the training, participants completed 5.89 (SD 4.43) modules and an average of 25.89 (SD 23.43) tasks and played 2.28 (SD 3.10) levels of the AAMT per module. The average error rate (incorrect responses) in the AAMT was 2.4%, suggesting that participants understood the training instructions. Participants spent 20.39 (SD 12.97) minutes playing the AAMT, representing 33% of the total time spent in the app. Regarding the intervention evaluation, participants in the intervention group were asked to evaluate the three major components of MT-Phoenix for their perceived helpfulness at postassessment using a Likert scale from 0 to 4. Evaluation results were above average, with high ratings for psychoeducation (mean 3.17, SD 0.72), the AAMT (mean 3.00, SD 0.53), and behavioral activation tasks (mean 3.17, SD 0.55).

## Discussion

This phase 2 pilot trial evaluated the preliminary effectiveness of a standalone gamified smartphone-based intervention combining AAMT and CBT principles in a sample of adults with elevated depression scores. Study results indicate a greater reduction of depressive symptom severity over the course of the intervention in the intervention group when compared to waitlist controls at posttreatment. Follow-up analyses indicate that effects were maintained over a period of 3 months after the completion of the intervention. In addition, the intervention group exhibited a significant increase in well-being at postassessment when compared to the control condition. These effects were also sustained through follow-up.

To the best of our knowledge, this is the first study to evaluate the effectiveness of a gamified intervention that combines CBT and AAMT techniques for the reduction of depressive symptoms. With regard to patient engagement, data analyses show a considerably high retention rate for MT-Phoenix (25/27, 93%, representing the percentage of participants that reused the app after the first use) when compared to studies showing that about 30% of individuals stop using smartphone-based interventions after the initial use [41]. Comparisons of adherence to smartphone-based depression interventions across studies indicate that participants trained with MT-Phoenix almost every other day (6.4/14 days, 46%), while intervention adherence was lower (39%) in a study that evaluated three smartphone-based interventions targeting depression [14]. However, comparisons are limited as smartphone-based interventions tend to differ on various parameters that have been shown to influence adherence such as interventional content, design, gamification elements, and use of reminders and notifications. Comparisons are further limited by varying study designs such as intervention periods, target populations, and use instructions. Moreover, the current study design did not include an experimental manipulation that tested the gamified smartphone-based intervention against a nongamified version. Thus, it cannot be concluded that the gamification elements used in MT-Phoenix were responsible for the engagement rates observed in this study. On the contrary, engagement with the intervention may have been influenced by participants with mild depression scores as such individuals have been shown to be more willing and able to use a self-directed intervention like MT-Phoenix [42].

To the best of our knowledge, this study is the first to evaluate the effectiveness of a gamified smartphone-based intervention targeting depressive symptoms using AAMT and principles of CBT. The observed effect sizes (*d*) were 1.02-1.41 for depression and 0.91 for well-being. Comparisons of effect sizes suggest that MT-Phoenix yields similar (well-being) and larger (depression) effects compared to smartphone-based interventions using gamified AAMT in combination with face-to-face CBT. Moreover, preliminary effects found in this pilot trial are superior to other standalone smartphone-based interventions for depression (*g*=0.56), as demonstrated in a meta-analysis [15]. Thus, MT-Phoenix is a promising low-threshold intervention for individuals with heightened depressive symptoms.

The findings presented in this paper have important theoretical and clinical implications. First, they provide evidence that a gamified smartphone-based intervention combining AAMT with CBT principles can reduce depressive symptoms. Second, this study is the first to show that a blended approach (smartphone-based intervention combined with face-to-face CBT) can be automated and delivered by a standalone smartphone-based intervention without any face-to-face contact with a mental health professional. Study results add to the growing body of literature suggesting that smartphone-based interventions have the potential to change the provision of mental health services profoundly, especially when interventions are scalable and easy to disseminate like the program under investigation. Third, this study demonstrates that a smartphone-based intervention targeting depression can engage participants to adhere to the intervention frequently. This finding is corroborated when comparing training data from this study with usage rates in other studies that tested smartphone-based interventions for depression.

Regardless of its merits, results from this study are subject to several limitations that need closer consideration. First, although similar interventions have been positively evaluated in pilot studies on body dissatisfaction [25], procrastination [26], and alexithymia [27], findings from this study provide preliminary evidence only for the particular program under investigation and thus cannot be easily generalized to other interventions combining AAMT and CBT. Hence, future studies should replicate these findings in other disorders or psychological problems. Second, as 30% of all participants received psychotherapy while participating in this study, results may have been influenced by factors other than the intervention under investigation. Further studies are needed to examine whether MT-Phoenix may be more appropriate as an adjunct to treatment-as-usual instead of a standalone intervention. Third, generalization of study results is limited as the smartphone-based intervention was made accessible for iPhone owners exclusively, the statistical power was low due to the rather small sample size, and the sample was homogeneous with regard to several sociodemographic variables. Thus, future studies should make MT-Phoenix available for other operating systems as well and should further examine the intervention in larger and more diverse samples. Fourth, inclusion of participants was performed on the basis of heightened depression scores rather than on the basis of a systematic diagnosis (eg, by using the Structured Clinical Interview for DSM-IV Axis I Disorders) [43]. We included participants who reported PHQ-9 depression scores of ≥5 to ensure testing of the intervention in individuals with a range of symptom severity and to allow for comparison between studies on smartphone-based interventions for depression that have focused predominately on participants with mild and moderate depressive symptoms. However, as we did not include an upper cutoff for study inclusion, participants with severe depressive symptoms were also included in the study. Approximately 30% of the study sample reported being in therapeutic treatment, thus hinting at the inclusion of patients with diagnosed depression. Future studies should try to replicate the findings in samples of patients that are more distinct with regard to depressive symptom severity. To this end, we are currently conducting a large multicenter clinical study that compares a gamified version of MT-Phoenix with a nongamified version in patients with a major depressive disorder diagnosis after release from inpatient treatment. Fifth, although the majority of participants in the treatment condition achieved clinically significant improvement, the intervention should be further improved to ensure positive outcomes for an even larger number of individuals. Sixth, future studies should attempt to further improve both study and intervention adherence. To this end, qualitative analyses of user data may be helpful to systematically identify and improve flaws in both the technology and content of the 1.1 version of the intervention used in this study. Seventh, the use of a nonactive waitlist control group may have led to an overestimation of treatment effects as waitlist designs have been discussed as possible nocebo conditions in the literature [44]. Despite the acknowledged limitations of research designs using nonactive control conditions, we decided to use an economic waitlist design in this pilot trial. Eighth, the high dropout rate before initiation of the intervention in the intervention group is another factor that has to be regarded with caution when interpreting the results of this study, especially in terms of the use of an intention-to-treat approach. Although uptake of automated online-based interventions with no personal contact between participants and study personnel is commonly observed as low in the literature [45], allocation to the control group and older age usually predict low uptake rates. Further observations are needed to identify possible reasons for this phenomenon beyond the aforementioned. Ninth, this pilot trial did not include an a priori power analysis to determine the sample size necessary for meaningful comparisons between treatment arms. Finally, despite the important role of approach-avoidance biases in depression, the current design prohibits assigning intervention effects to the AAMT. Further dismantling or comparison studies are needed to ascribe intervention effects to distinct techniques used in MT-Phoenix. Another possible solution to this limitation is to systematically measure approach and avoidance tendencies at both pre- and posttraining.

## Appendix

Multimedia Appendix 1 App screenshots.

Multimedia Appendix 2 Stimuli examples.

Multimedia Appendix 3 CONSORT flow diagram.

Multimedia Appendix 4 CONSORT-eHEALTH checklist (V 1.6.1).

## Abbreviations

AAMT: approach-avoidance modification training
ADS: Allgemeine Depressions-Skala
ANCOVA: analysis of covariance
CBM: cognitive bias modification
CBT: cognitive-behavioral therapy
MT-Phoenix: mentalis Phoenix
PHQ-9: Patient Health Questionnaire-9
WHO-5: 5-item World Health Organization Well-being Index
