# Supplementary figures and images for: A Gamified Smartphone-Based Intervention for Depression: Randomized Controlled Pilot Trial
Source: JMIR Ment Health. 2021 Jul 20;8(7):e16643. doi: 10.2196/16643 (PMC8335612; doi:10.2196/16643)

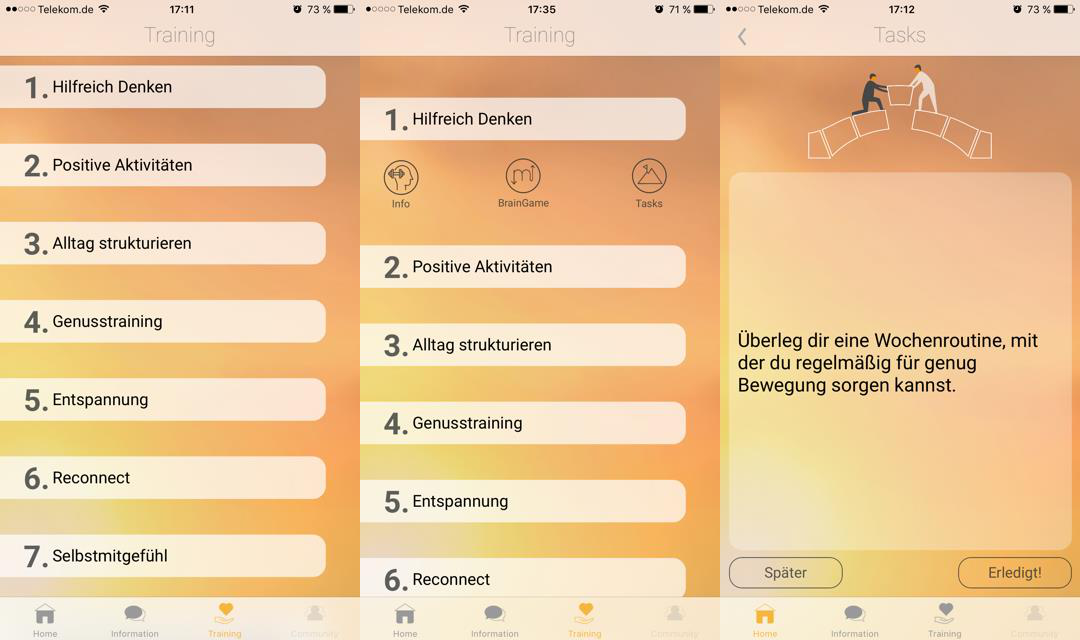

Supplement: Multimedia Appendix 1 [file mental_v8i7e16643_app1.png]

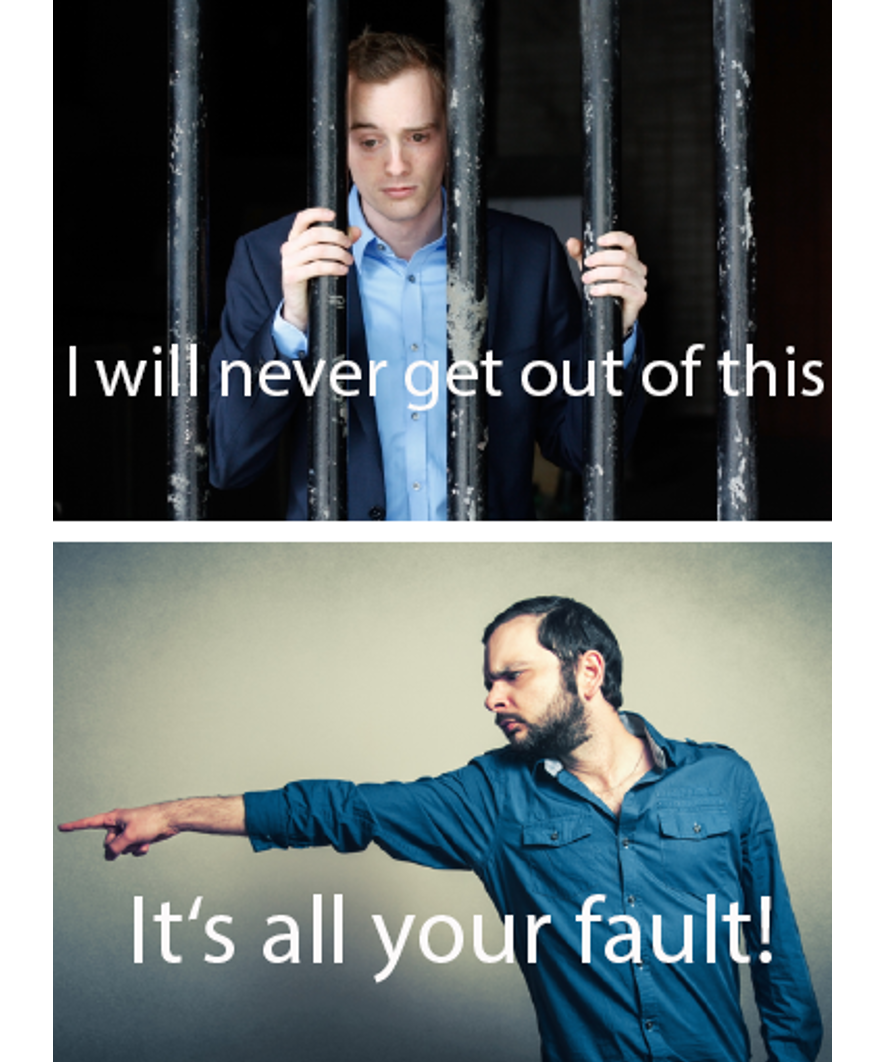

Supplement: Multimedia Appendix 2 [file mental_v8i7e16643_app2.png]

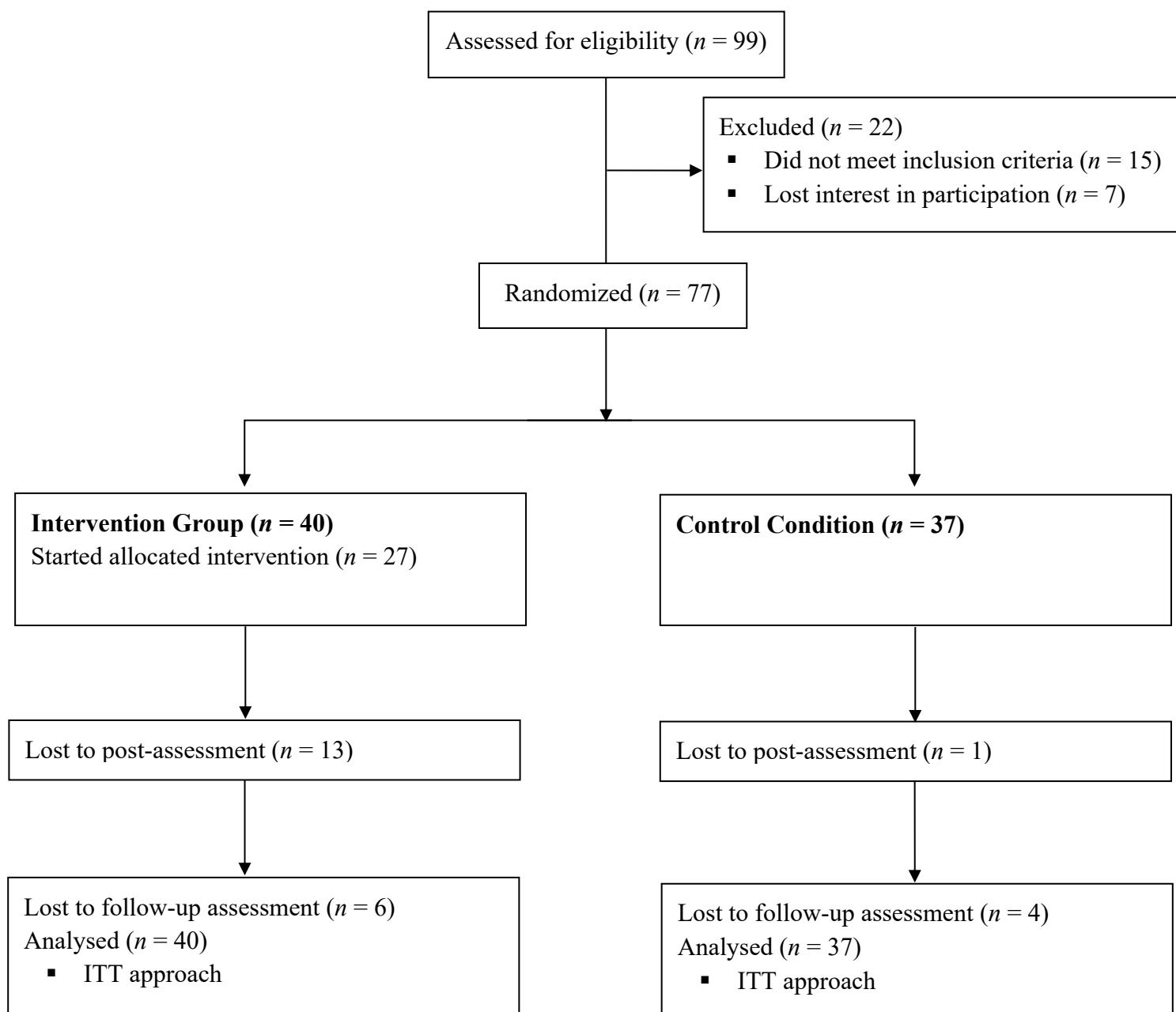

Supplement: Multimedia Appendix 3 [file mental_v8i7e16643_app3.pdf]
